# Carbonic Anhydrase and Biomarker Research: New Insights

**DOI:** 10.3390/ijms24119687

**Published:** 2023-06-02

**Authors:** Maria Giulia Lionetto

**Affiliations:** 1 Department of Biological and Environmental Sciences and Technologies (DiSTeBA), Salento University, 73100 Lecce, Italy; giulia.lionetto@unisalento.it; 2 National Biodiversity Future Center (NBFC), 90133 Palermo, Italy

Carbonic anhydrase (CA) is a widespread metalloenzyme with eight genetically distinct families catalyzing the reversible hydration of CO_2_ to HCO_3_^−^ and H^+^. CA plays a pivotal role in several physiological processes, such as pH regulation, respiration, electrolyte transport, metabolism, bone resorption, and calcification.

For many decades CA isozymes have been recognized as being involved in several pathological processes, giving rise to the research on CAs as useful diagnostic/prognostic biomarkers in the clinical field. Moreover, the sensitivity of several carbonic anhydrase isoforms to environmental chemical contaminants in humans and wildlife has opened new perspectives for the potential application of alterations in carbonic anhydrase activity and expression as pollution biomarkers [1,2,3,4].

A biomarker represents a molecular, biochemical, or cellular alteration detectable in a biological system as an indicator of biological processes, pathological conditions, susceptibility, or response to exposure. They are valuable tools in a wide range of fields, including clinical application, drug discovery, and human and environmental biomonitoring. The biomarker research in the last two decades has received great attention in several research fields, as indicated by the growing trend of publications and patents. In this stimulating research area, the study of carbonic anhydrase is of relevance thanks to its wide distribution, its key role in a number of physiological processes, its involvement in several pathological conditions, and its sensitivity to chemical pollutants.

The Special Issue entitled “Carbonic Anhydrase and Biomarker Research 2020” in the *International Journal of Molecular Sciences* provides emerging knowledge into the research field of carbonic anhydrase as a promising biomarker in several scientific areas, from human health to environmental sciences. The original publications collected in the Issue are representative of the multifaceted aspects of carbonic anhydrase in biomarker research and open new perspectives for the transfer of findings in basic research on this important enzyme into novel applications.

In the research field of biomarker discovery and development for human health, great interest is devoted to isoforms IX and XII of CA due to their involvement in cancer [1]. CAIX and CA XII are involved in the tolerance of cancer cells to hypoxia and acidosis, which characterize the microenvironment of solid tumors. CAIX is highly expressed in several types of tumors, including breast, lung, ovarian, bladder, oral squamous cell, astrocytic, and hepatocellular carcinoma, while CA XII is observed in kidney, colorectal, breast, gastric, and brain tumors [5,6].

Carbonic anhydrase IX is a transmembrane glycoprotein constituted by a brief cytoplasmic region, a transmembrane helix, an extracellular domain, and a N-terminal proteoglycan-like domain (PG). Its aberrant expression is found in several human cancer types and is recognized as a marker of tumor hypoxia. CAIX is characterized by a high catalytic efficiency and hypoxia-related expression and is typically related to a poor prognosis. Four papers on the issues focus on CAIX analyzing different aspects of the involvement of this protein in the biomarker research field. The works of Koruza et al. [7] and Simko et al. [8] provide new insight into the molecular structure of the protein with implications for its role in cancer. In particular, Koruza et al. [7] reported the crystal structure of the catalytic domain of CAIX and small-angle X-ray scattering and mass spectrometry of the extracellular region including the PG domain, knowledge of which is limited to date. The extracellular domain and its dynamics, including the cleavage and shedding of the PG domain observed by the authors, could have physiological relevance in the role of CAIX in cancer progression. Simko et al. [8] highlighted the C-terminal Ala459 residue as an essential element for the catalytic activity of CAIX and showed that a structural change at the 459 position decreases the CA enzymatic activity. Moreover, the authors demonstrated the intracellular interaction of CAIX with the cytoplasmic protein PIMT, known for its protein repair function, and identified the Ala459 residues as a mediator of this interaction. The physical interaction between CAIX and PIMT induced by tumor hypoxia is suggested to act as a novel inside-out modulation of CAIX activity and pH regulation.

The involvement of CAIX in breast cancer and renal cancer biomarker research is deepened by the studies of Gibadulinova et al. [9] and Courcier et al. [10], respectively. Gibadulinova et al. [9] studied the effects of the silencing of CA9 in breast cancer cell lines. The authors found that the levels of members of the let-7 family of miRNAs were increased by CAIX depletion in hypoxic breast cancer cells accompanied by a decrease in the reprogramming factor LIN28, NF-κB activity, and glycolytic metabolism. Moreover, CAIX knockout cells also showed cancer stem-cell-associated markers, such as Homeobox protein NANOG and Aldehyde dehydrogenase isoform 1. On the other hand, the overexpression of CAIX induced the enhancement of LIN28, ALDH1, and NANOG. The authors concluded that CAIX drives the regulation of the LIN28/let-7 axis augmenting the glycolytic metabolism and enhancing the expression of stem cell markers during the adaptation to hypoxia and acidosis of cancer cells mediated by CAIX. Courcier et al. [10], in their review, analyzed the current literature on the relationship between CAIX expression and renal cell carcinoma, focusing on the role of CAIX as a useful marker in this pathology. The importance of CAIX in renal cancer as a biomarker is well recognized, particularly for the clear cell subtype, which represents the most diffused form of renal cancer cells. As outlined by the authors, the usefulness of CAIX immunohistochemical analysis as a biomarker is well recognized, while the development of molecular imaging is still exploratory and encourages future studies in the field.

In the last few years, various sulfonamide derivative compounds have been studied as anticancer drugs due to their ability to inhibit the activity of human tumor-associated carbonic anhydrase isoforms CAIX and CAXII. In the work of Mikulová et al. [11], different synthetic pathways for the series of 1,3,5-triazinyl-aminobenzenesulfonamide conjugates with amino acids were reported. Many of these compounds represent highly potent and selective inhibitors of human CA, showing great potential for further investigation in the oncological field.

Another promising field of research in the diagnosis and treatment of cancer is represented by monoclonal and recombinant antibodies constructed to interact with membrane proteins involved in the carcinogenesis process. The work of Stravinskiene et al. [12] provides novel monoclonal antibodies against the recombinant extracellular domain of CAXII. CAXII is detected in various tissues, while in cancer and tumors, its expression is several-fold higher, being identified as a cancer marker. Its expression is regulated by hypoxia and estrogen receptors [13]. CAXII helps the survival of tumor cells under hypoxic conditions; therefore, it can represent a good target for antibody-based therapy. Antibodies are favorable tools for CAXII-related cancer research and immunotherapy. Stravinskiene et al. [12] described novel murine monoclonal antibodies constructed against cancer-associated enzyme CAXII, useful for CAXII research and for diagnostics.

Zn^2+^ is the fundamental cofactor of the catalytic activity of all CA isoforms; its role is to promote the deprotonation of H_2_O with the production of OH′, which, in turn, can nucleophilically attack the carbonyl group of CO_2_ to convert it into HCO_3_^−^ [14]. Therefore, the proper acquisition of Zn^2+^ by apoCA during its maturation is a fundamental step in the genesis of the enzyme, which is, to date, very poorly investigated. Metallothioneins represent a major Zn^2+^ storage protein in the cell and are key in activating Zn-dependent enzymes, including CA [15]. In their review, Wong et al. [16] contributed to improving the knowledge in the field of CA metalation. They described a series of experiments regarding the metalation of carbonic anhydrase based on the Zn^2+^ transfer from Zn-MT to apo-CA and concluded that this process likely involves protein–protein interactions that deserve further investigation. The authors hope for advancements in mass spectrometry, useful for providing the methodological approach to investigate this intriguing field of research.

In recent years, CA has also been found to have implications for neurological and psychiatric disorders. Noninvasive in vivo magnetic resonance spectroscopy (MRS) has emerged as a useful tool for investigating the role of carbonic anhydrase in many brain disorders as well as in monitoring treatment. Tomar and Shen [17] reviewed the methodological aspects of in vivo ^13^C magnetization (saturation) transfer magnetic resonance spectroscopy for application to the in vivo measurement of CA catalytic reaction in brain disorders where biopsies are not practicable.

The interest in CA in the biomarker research field is not limited to clinical applications but also embraces other fields of research such as environmental sciences. In recent years, the increasing interest in developing environmental “diagnostic” tools for the early warning detection of pollution and its impact on wildlife and humans gave impetus to developing pollution biomarkers useful for environmental biomonitoring and risk assessment. In this research field, plenty of evidence emerged on the effect of pollutants on carbonic anhydrase activity and expression, offering new perspectives in the application of this enzyme as a biomarker in environmental biomonitoring [2,3]. The review of Lionetto et al. [18] highlights the involvement of carbonic anhydrase research in the field of pollution biomarker discovery. In particular, the work analyzes and discusses the recent literature on the sensitivity of CA to pesticides in humans and wildlife, providing new insight for the development of novel CA-based pesticide biomarkers for applications in various scientific areas, from occupational and environmental medicine to environmental biomonitoring.

In conclusion, the study of CA represents a fruitful and multifaceted research arena for developing novel approaches for biomarker development and implementation; it offers a wide range of perspectives in several fields, from clinical research to environmental sciences.
